# Alveolar Abcess

**Published:** 1866-08

**Authors:** A. Moore


### ALVEOLAR ABCESS.

[Read before the Indiana State Dental Association.]

BY DR. A. MOORE.

The pathologist is able to trace several distinct stages of this disease from its incipiency to its termination of final cure. Its first, which is increased vascular action, may be denominated a strong determination on the part of the vascular system to do more than its capacity will allow, which fact results in congestion, a true disease, characterized by an increased amount of dark blood passing sluggishly through the veins and capillaries until stagnation is the final result.

Inflammation of the pulp which may arise from a variety of causes, is most generally induced to a considerable extent; in this stage of the disease of the periosteum it is modified somewhat by the diathesis of the patient; being most likely to supervene in strumous and scrofulous habits. What ever may be the cause, the symptoms attending it are generally the same. At first there is a sensation of uneasiness and a feeling of fullness, with a desire to press the teeth together, and unless arrested the pain increases, becoming dull, heavy, and continuous, being an effort of the distended vessels to void themselves of their contents, by the transudation of the watery fluid of the blood into the surrounding tissue, causing it in common parlance to "swell or puff up." The treatment at this stage is local depletion with the lance or leech, accompanied with anti-phlogistic remedies; but prior to this it may be expedient to use cold application, as ice held in contact with the gums, which acts as an astringent, contracting the blood vessels and diminishing the quantity of the blood to that particular part Iodine has a very salutary effect when applied over the end of the root involved; the nitrate of potash introduced into the pulp cavity acts as an arterial sedative, and when followed up by the anti-phlogistics the disease is quite certain to succumb.

But should it pass on to violent inflamation, which is characterized by an increase of temperature and more violent pain, spreading to the surrounding tissues, involving the entire side of the face, the bloodvessels are increased in size with the quantity and rapid motion of the blood, with a tendency to stagnation at points caused by the clogging up of the veins and capillaries by the red corpuscles which are more closely packed in consequence of the watery fluid passing out. In an advanced stage of inflammation the swelling is due to an effusion of lymph, and an abnormal growth from its organization, which cause the tooth to rise in the socket in consequence of the density of the contiguous parts. As the disease advances the pain is of a pulsating character, owing to the compression of the nerves and exalted sensibility. So much so that great pain is felt when the tooth is touched. The heat in the inflamed part rises above the normal standard caused by the waste and oxidation of the tissues.

When the disease has reached this stage two kinds of treatment are indicated: first, to induce resolution by the course of treatment adopted for congestion: second, if this fails, hasten to bring about suppuration, which is done by the use of hot applications, roasted figs should be applied to the gums, and a poultice made of hops and laudanum to the cheek as an anodyne and soporific,

The effusion of lymph which always accompanies inflammation as it advances, is of two kinds, plastic or fibrous, and the corpuscular. The first, according to Atkinson, is the true lymph, and capable when in contact with living healthy tissues and in the hands of a skillful surgeon, of organization and giving back to the patient parts quite as perfect as ever.

The corpuscular or second kind is not capable of organization, and instead of fibers contains exudation corpuscles; which evidently must undergo one more process, for no visible change has yet taken place in their color—which does follow after the periosteum has become detached, and a sac is formed for the reception of pus, which is the next change. Whether this quality of lymph is dead, matters very little, as it is not susceptible of being again appropriated, or influenced by the life-giving principle, and organized again into living tissue. It must follow, then, that this condition of pus cells is best suited to be thrown to the surface and cast off by nature, not being able to discern any disposition upon her part to relieve herself of it, until it has assumed this character. As soon as it is formed it immediately seeks an outlet, and usually takes the most direct course to the surface, particle after particle giving way by pressure or the degenerating effects of pus cells in contact with the living tissues until it reaches the surface, when relief is immediately felt, and the swelling in the face gradually subsides.

After the puss is discharged and no treatment instituted, a chronic form will ensue, and the sac will again fill and discharge, in frequency according to the habit of the individual. There are various modes of treatment at this stage of the disease. The most efficient of which is to remove the exciting cause which is most likely to be the dead pulp or gaseous matter confined within ~~the~~ pulp cavity. This should be thoroughly cleansed, and if the fistulous opening still remaining opposite the diseased root, pump creosote and tincture of iodine in proportion of three of the former to one of the latter, through the apex of the root by means of a broach wrapped with cotton and dipped into the drug until the cauterizing effects of it are seen at the mouth of the fistula.

A few days of treatment of this kind and the sac is broken up, and will be speedily absorbed, when the periosteum will again become united to the bone, by the plastic lymph being thrown in the form of adhesive plasm. When the root is ready for filling, which should not be overlooked if you would not have your skill and labor in vain.

